# Association of Human Leukocyte Antigen Alleles and Nevirapine Hypersensitivity in a Malawian HIV-Infected Population

**DOI:** 10.1093/cid/cit021

**Published:** 2013-01-29

**Authors:** Daniel F. Carr, Mas Chaponda, Andrea L. Jorgensen, Elena Cornejo Castro, Joep J. van Oosterhout, Saye H. Khoo, David G. Lalloo, Robert S. Heyderman, Ana Alfirevic, Munir Pirmohamed

**Affiliations:** 1 Department of Molecular and Clinical Pharmacology, University of Liverpool, United Kingdom; 2 Malawi-Liverpool-Wellcome Trust Clinical Research Programme, Blantyre, Malawi; 3 Department of Biostatistics, University of Liverpool, United Kingdom; 4 Department of Medicine, College of Medicine, University of Malawi, Blantyre; 5 Liverpool School of Tropical Medicine, United Kingdom

**Keywords:** nevirapine, hypersensitivity, Stevens-Johnson syndrome, human leukocyte antigen, genetics

## Abstract

***Background.*** The nonnucleoside reverse transcriptase inhibitor nevirapine is the cornerstone of treatment for human immunodeficiency virus (HIV) in many sub-Saharan African countries. However, nevirapine is associated with a 6%–10% risk of developing a hypersensitivity reaction, with different phenotypes, including the blistering conditions Stevens-Johnson syndrome (SJS) and toxic epidermal necrolysis (TEN). Our aim was to identify predictive human leukocyte antigen (HLA) markers that are associated with nevirapine hypersensitivity.

***Methods.*** We identified 117 HIV-infected Malawian adults with nevirapine hypersensitivity (15 drug-induced liver injury [DILI], 33 SJS/TEN, 20 hypersensitivity syndrome, and 46 nevirapine-induced rash plus 3 with both DILI and SJS phenotype) and 155 age-, sex- and ethnicity-matched nevirapine-exposed controls. HLA typing for 5 loci *(A*, *B*, *C*, *DRB1*, and *DQB1*) was undertaken using a sequence-based high-resolution protocol. Logistic regression analysis included CD4^+^ cell count as a covariate.

***Results.*** HLA-C*04:01 was found to markedly increase the risk for SJS (odds ratio [OR] = 17.52; 95% confidence interval, 3.31–92.80) and all hypersensitivity phenotypes (OR = 2.64; 95% CI, 1.13–6.18) when compared to the baseline rare allele group in a binary logistic regression model. The OR for absolute risk of SJS/TEN associated with carriage of HLA-C*04:01 was 5.17 (95% CI, 2.39–11.18). Positive predictive value was 2.6% and negative predictive value was 99.2%. In addition, a number of alleles within the *HLA-DQB1* loci protected against nevirapine-induced hypersensitivity phenotypes.

***Conclusions.*** Our study has identified HLA-C*04:01 carriage as a risk factor for nevirapine-induced SJS/TEN in a Malawian HIV cohort. Validation of these findings in a larger cohort of patients and mechanistic investigation of the pathogenesis are required.

## Abstract (precis)

Human leukocyte antigen genotyping of 272 Malawian HIV patients receiving nevirapine-containing regimens (of whom 117 had nevirapine hypersensitivity) has shown that HLA-C*04:01 increases the risk of Stevens-Johnson syndrome/toxic epidermal necrolysis, with an odds ratio of 5.17 (95% confidence interval, 2.39–11.18).

The nonnucleoside reverse-transcriptase inhibitor nevirapine is widely used as a first-line treatment of human immunodeficiency virus (HIV) [1] infection in developing countries because of its low cost. Nevirapine is usually given in combination with 2 nucleoside reverse-transcriptase inhibitors (stavudine or zidovudine and lamivudine).

Though effective [2], nevirapine causes hypersensitivity in 6%–10% of patients [3, 4], which can manifest clinically in various ways from nevirapine-induced rash (without any systemic manifestations) to severe blistering skin reactions such as Stevens-Johnson syndrome and toxic epidermal necrolysis [5] (1–2 per 1000 exposed individuals [6]). Extracutaneous involvement typically manifests as hepatotoxicity [7]. Reactions most commonly manifest within the first 6 weeks of starting therapy.

Immunogenetic factors, including a number of human leukocyte antigen (*HLA*) alleles, have been previously identified as risk factors for hypersensitivity reactions to the antiretroviral abacavir [8] and many other classes of drugs, including the antiepileptic drug carbamazepine [9, 10] and the antigout drug allopurinol [11]. Research has also focused on immunogenetic risk factors for nevirapine hypersensitivity, identifying the HLA-DRB1*01:01 (whites [12–14]), HLA-C*04 (Thai [15], Chinese [16], and blacks [14]), HLA-C*08 (Japanese [17]) and HLA-B*35:05 (Thai [14, 18]) as risk alleles (Table 1).

**Table 1.** Previously Reported Human Leukocyte Antigen Allele Associations With Nevirapine Hypersensitivity

Phenotype [Reference]	Population	Cases/Controls (No.)	Risk			Protective		
			*HLA* Allele	OR (95% CI)	*P* Value	*HLA* Allele	OR (95% CI)	*P* Value
1) HSRs (12 DILI) [16]	Han Chinese	32/71	HLA-C*04	3.61 (1.13–11.49)	.03	HLA-DRB1*15	0.34 (.12–.99)	.049
2) Rash [15]	Thai	39/60	HLA-C*04	3.18 (1.33–8.63)	.009	HLA-C*03	0.27 (.09–.82)	.01
3) Isolated rash [13]	White	6/15	HLA-DRB1*01	70.0 (3.65–1342.66)	.004^a^			
4) Rash/systemic with associated hepatitis [12]	White (Australia)	15/64	HLA-DRB1*01	4.77 (1.55–14.73)	.01			
5) All HSRs	Sardinian	13/36	HLA-C*08/B*14	14.57 (2.42–87.73)	.004^a^			
3 systemic with rash and/or liver toxicity								
5 extensive skin rash								
5 isolated hepatotoxicity [40]								
6) Rash [18]	Thai	143/181	HLA-B*35:05	18.96 (4.87–73.44)	4.9 × 10^−8a^	HLA-C*07:02	0.40 (.20–.78)	.0067
7) All HSRs	Japanese	41/41	HLA-C*08	6.19 (1.18–32.40)	.03			
8 rash; 3 rash + fever; 1 DILI [17]								
8) Cutaneous HSRs [14]	Mixed	175/587	HLA-C*04	2.51 (1.73–3.62)	6.7 × 10^−7a^			
	Black	27/77		5.17 (2.01–13.30)	9.5 × 10^−4a^			
	Asian	71/233		2.55 (1.41–4.60)	.0028^a^			
	Asian	71/227	HLA-B*35/C*04	18.34 (5.10–65.99)	2.4 × 10^−7a^			
	Thai	52/173		13.49 (3.56–52.20)	3.4 × 10^−5a^			
9) Isolated hepatotoxicity [14]	White	57/277	HLA-DRB1*01	3.02 (1.66–5.49)	5.7 × 10^−4a^			
10) Rash, 4 SJS, 3 with hepatitis [41]	Indian	40/40	HLA-B*35	3.38 (1.54–7.41)	.003	HLA-B*8	0.29 (.12–.71)	.008

Where odds ratios and 95% CIs are not reported, a 2 × 2 χ^2^ test was performed on the data available.

Abbreviations: CI, confidence interval; DILI, drug-induced liver injury; HLA, human leukocyte antigen; HSR, hypersensitivity reaction; OR, odds ratio; SJS, Stevens-Johnson syndrome.

^a^ Denotes reported associations which withstood correction for multiple testing (*P*_corrected_ < 0.05).

To date, little is known regarding genetic risk factors for nevirapine-induced hypersensitivity in sub-Saharan African HIV-infected populations. Using a cohort of carefully phenotyped Malawian patients, we have undertaken high-resolution sequence-based genotyping to determine whether alleles in 5 loci in the class I and II major histocompatibility complex (MHC) regions on chromosome 6 (*HLA- DRB1*, *DQB1*, *A*, *B*, or *C*) are predisposing factors for nevirapine hypersensitivity.

## PATIENTS AND METHODS

### Patients

Between March 2007 and December 2008, we prospectively recruited 1117 antiretroviral-naive adult patients from the outpatient clinic at the Queen Elizabeth Central Hospital, Blantyre, Malawi. At time of recruitment, this clinic had approximately 10 000 patients registered as having started on antiretroviral therapy since 2004. Patients were self-reported black African; were older than 16 years; and gave informed consent approved by the research ethics committees at the College of Medicine Research and Ethics Committee, Malawi, and Liverpool School of Tropical Medicine. Patients presenting with jaundice at baseline were excluded.

Patients commenced antiretroviral therapy as recommended by the World Health Organization eligibility criteria at the time of recruitment. All patients were diagnosed as clinical stage 3/4 or had a CD4^+^ count <250 cells/µL; commenced preparations, which contained a fixed dose of nevirapine, lamivudine, and stavudine; and were followed up for 26 weeks. Clinical and laboratory parameters including CD4^+^ count and liver function tests were monitored at 0, 6, 14, and 18 weeks.

The study was a nested case-control study; however, because of the low incidence of hypersensitivity in the prospectively recruited cohort, an additional 177 patients attending the same outpatient clinic who developed nevirapine hypersensitivity were also recruited, either prospectively (n = 149) or identified from patient records retrospectively (n = 28). Of 177 patients, 65 were excluded owing to insufficient DNA quality and quantity.

Careful clinical assessment of all patients was undertaken to identify and characterize the hypersensitivity reactions, using the Naranjo causality assessment tool [19, 20]. Phenotypes were retrospectively reviewed independently by a dermatologist using both clinical data and photographs. These were defined as:

- Nevirapine-induced rash (NIR): widespread maculopapular rash without systemic manifestations and getting worse on treatment continuation.
- Hypersensitivity syndrome (HSS; also known as *drug reaction with eosinophilia and systemic symptoms* or *drug-induced hypersensitivity syndrome*): widespread rash and systemic manifestations such as fever, cough, or abnormal liver function tests.
- Stevens-Johnson syndrome (SJS): extensive rash with the involvement of at least 2 mucous membranes or blistering eruptions affecting <10% of body surface area.
- Toxic epidermal necrolysis (TEN) [5]; blistering rash affecting >30% of body surface area and mucous membrane involvement as per SJS [21]. Blistering between 10% and 30% of body surface area was termed *overlap syndrome*.
- Drug-induced liver injury (DILI) [7]: jaundice and abnormal alanine aminotransferase level.

Patients meeting criteria for drug-induced reactions had nevirapine withdrawn in accordance with international guidelines. It is important to note that as part of the Malawian treatment guidelines, liver function tests are not routine, and therefore, abnormal tests without clinical jaundice did not fulfill criteria for treatment cessation and were not included as cases. Furthermore, some patients who developed transient nonsevere rash without systemic symptoms underwent close observation, were treated continuously with rash resolution, and again were not classified as cases.

Control patients (n = 155) were identified from the prospective cohort and followed up for at least 6 months while taking nevirapine without developing any signs of hypersensitivity. Cases and controls were matched by age and sex, and were also from the same region of Malawi.

### DNA Extraction and High-Resolution Sequence-Based HLA Typing

DNA was extracted from whole blood using a salt precipitation protocol. High-resolution, sequencing-based *HLA* typing of 5 loci (*HLA-A*, *B*, *C*, *DRB1*, and *DQB)* was undertaken by Histogenetics (Ossining, New York). Sequencing data files were analyzed using Histogenetics’ proprietary analysis software (Histomatcher and HistoMagic) for HLA genotype calling. Allele assignments are based on IMGT/HLA Database release version 2.21.0, dated April 2008 (http://www.ebi.ac.uk/imgt/hla/).

### Statistical Analysis

Sample size calculations were performed assuming that a 10% background frequency of an *HLA* allele would provide 80% power (α = .05) to detect an odds ratio (OR) of 3.0 (and 90% power to detect an OR of 3.4). We included all patients with hypersensitivity in the analysis. Subgroup analyses were performed for all phenotypes, (DILI, SJS/TEN, HSS, NIR) where we compared the frequency of *HLA* alleles in patients with nevirapine-induced adverse reaction with the frequency in tolerant individuals.

Nongenetic factors identified a priori as being of importance, such as CD4^+^ count, were first tested univariately for association with hypersensitivity reaction (all cases) using the Student *t* test. The distribution of CD4^+^ count was skewed, and a square-root transformation was used to achieve normality. CD4^+^ count for 20 cases was missing, and these observations were substituted by the mean-transformed CD4^+^ count for all cases where CD4^+^ count was observed. Differences in frequencies of alleles in individual *HLA* locus between tolerant patients and each of the hypersensitivity phenotypes were determined from 2 × N contingency tables using a χ^2^ test within the CLUMP software package (http://www.smd.qmul.ac.uk/statgen/dcurtis/lc/clump.html). To determine association with specific alleles within hypersensitivity-linked *HLA* loci, 2 logistic regression models were fitted. The first included covariates representing the nongenetic factors identified from univariate analysis (*P* < .05). The second included a covariate to represent *HLA* alleles assuming a dominant mode of inheritance. Rare alleles were grouped into a single allele category and, because this represented the largest category, it was assumed to be the baseline allele category for the purpose of regression modeling. To assess for significance of the genetic locus, a likelihood-ratio test was undertaken comparing the models and the *P* value was recorded. Analyses were undertaken in R version 2.13.0. To account for multiple comparisons (5 phenotypes and 5 loci), we used the false-discovery rate method within the “p.adjust” function of R. The *HLA* multiple locus haplotypes were generated using PyPop 0.7.0 software [22].

A random-effects OR meta-analysis of pooled data from our study and previously published data was undertaken using StatsDirect version 2.6.8 (StatsDirect Ltd, Atrincham, UK).

## RESULTS

From the prospective cohort (n = 1117), 57 patients developed hypersensitivity (5.1%), of whom 31 were successfully *HLA*-typed. Of the 149 supplementary hypersensitive patients, 86 were *HLA*-typed, giving a total of 117 HLA-typed hypersensitive patients (15 DILI, 33 SJS/TEN, 20 HSS, and 46 NIR, plus 3 individuals with the DILI and SJS/TEN phenotype). One control sample failed *HLA* typing, leaving 154 HLA-typed drug-tolerant controls. The overall HLA-allele call rates were 182 of 271 (67%) for DRB1*; 241 of 271 (89%) for DQB1*; and 296 of 271 (99%) for *A*, *B*, and *C*.

A summary of the *HLA* allele frequencies for each of the phenotypes and controls is provided in Supplementary Table 1.

Median CD4^+^ cell count at the start of antiretroviral therapy was 235 cells/µL (interquartile range [IQR], 128–424 cells/µL) in cases and 166 cells/µL (IQR, 83–250 cells/µL) in controls. This represented a statistically significant difference; thus, CD4^+^ cell count was adjusted for in the analyses of association with genetic loci.

We undertook χ^2^ analyses in CLUMP focusing on the association of each locus with the different phenotypic manifestations (Table 2). After correction for multiple comparisons, we identified *HLA-DQB1* as the only significant (*P*_corrected_ < .05) *HLA* locus for nevirapine-induced hypersensitivity, when all phenotypes were combined, and with SJS/TEN specifically. The locus-specific analysis provided an indication that the *HLA-DQB1* region protected against nevirapine hypersensitivity. Given the high degree of linkage disequilibrium in the MHC, and the multiple alleles present within each locus, we then undertook an analysis of the individual *HLA* alleles (Table 3). Consistent with the locus-specific data, a number of *HLA-DQB1* alleles were found to protect against nevirapine hypersensitivity when compared to the “rare allele” group. These included 6 different DQB1* alleles (*02:01G*, *03:02:01*, *05:01:01*, *06:02*, *06:03:01*, and *06:09*) associated with a decreased risk of all hypersensitivity reactions with ORs ranging from 0.17 (95% CI, .05–.6) to 0.41 (95% CI, .18–.96). DQB1*05:0101 was protective for SJS (OR = 0.11 [95% CI, .02–.56]) and HSS (OR = 0.17 [95% CI, .04–.80]); and *06:02* for DILI (OR = 0.09 [95% CI, .01–.52]) and HSS (OR = 0.16 [95% CI, .04–.75]). One DRB1* allele (*15:03*) protected against DILI (OR = 0.08 [95% CI, .01–.59]).

**Table 2.** Logistic Regression Analysis for 5 Human Leukocyte Antigen Loci in All Patients With Nevirapine-Induced Hypersensitivity

Locus	*P* Value				
	All HSR	SJS/TEN	DILI	NIR	HSS
*HLA-A*	894	**.015**	.429	.672	.135
*HLA-B*	111	.160	.068	.478	.066
*HLA-C*	**036**	**.014**	.179	.489	.564
*HLA-DQB1*	002*	.003*	**.006**	.148	**.030**
*HLA-DRB1*	137	.123	**.018**	.455	.477

*P* values are derived from a likelihood ratio test comparing a logistic regression model both with and without a covariate representing the alleles observed at the genetic locus. Statistically significant findings (*P* < .05) are indicated in bold while associations withstanding correcting for multiple comparisons (false discovery rate *P* < .05) are indicated by an asterisk (*).

Abbreviations: DILI, drug-induced liver injury; HLA, human leukocyte antigen; HSR, hypersensitivity reaction; HSS, hypersensitivity syndrome; NIR, nevirapine-induced rash; SJS, Stevens-Johnson syndrome; TEN, toxic epidermal necrolysis.

**Table 3.** Association of Specific Human Leukocyte Antigen Alleles With Nevirapine-Induced Hypersensitivity Phenotypes Compared to the Baseline “Rare Allele” Group

Loci	Allele	Odds Ratio (95% CI)			
		All HSR (n = 116)	SJS/TEN (n = 36)	DILI (n = 18)	HSS
HLA-A*	*01:01*			n/a	
	*02:01*			1.63 (.45–5.93)	
	*02:05*			1.19 (.18–7.97)	
	*03:01*			3.11 (.62–15.59)	
	*23:01*			.52 (.12–2.30)	
	*29:02:01*			1.81 (.42–7.71)	
	*30:01*			1.04 (.28–3.84)	
	*30:02*			.59 (.16–2.22)	
	*34:02*			3.59 (.89–14.57)	
	*36:01*			2.23 (.51–9.75)	
	*66:01*			n/a	
	*68:01*			n/a	
	*68:02*			2.80 (.81–9.72)	
	*74:01*			.36 (.06–2.14)	
HLA-C*	*02:10*	.56 (.21–1.49)	1.38 (.22–8.72)		
	*03:02*	1.03 (.24–4.4)	3.51 (.25–49.79)		
	*03:03*	1.42 (.31–6.43)	1.59 (.08–31.76)		
	*03:04:02*	1.43 (.46–4.41)	4.85 (.8–29.54)		
	*04:01*	**2.64 (1.13**–**6.18)**	**17.52 (3.31**–**92.8)**		
	*06:02*	.67 (.27–1.67)	1.25 (.18–8.57)		
	*07:01*	2.04 (.81–5.14)	4.00 (.73–21.84)		
	*07:02*	1.08 (.33–3.55)	3.06 (.35–26.72)		
	*07:04*	1.40 (.36–5.42)	6.68 (.61–72.57)		
	*08:02*	.99 (.37–2.68)	2.40 (.39–14.8)		
	*12:03*	.82 (.19–3.6)	1.69 (.15–19.67)		
	*16:01:01*	.90 (.29–2.78)	3.78 (.52–27.35)		
	*17:01*	1.43 (.56–3.63)	5.95 (.94–37.55)		
	*18:01*	.92 (.34–2.49)	2.02 (.29–14.08)		
	Allele	All HSR (n = 106)	SJS/TEN (n = 35)	DILI (n = 14)	HSS (n = 17)
HLA-DQB1*	*02:01G*	**.41 (.18**–**.96)**	.98 (.30–3.24)	**.08 (.01**–**.61)**	.26 (.06–1.19)
	*03:01G*	1.12 (.45–2.75)	2.26 (.60–8.54)	1.13 (.23–5.69)	.83 (.19–3.57)
	*03:02:01*	**.32 (.08**–**.94)**	.62 (.09–4.29)	.40 (.03–5.25)	n/a
	*04:02*	.39 (.13–1.16)	.67 (.12–3.59)	.14 (.01–1.81)	.60 (.10–3.48)
	*05:01:01*	**.27 (.12**–**.63)**	**.11 (.02**–**.56)**	.29 (.06–1.45)	**.17 (.04**–**.80)**
	*06:02*	**.30 (.13**–**.72)**	.87 (.22–3.45)	**.09 (.01**–**.52)**	**.16 (.04**–**.75)**
	*06:03:01*	**.17 (.05**–**.60)**	.27 (.04–1.80)	.26 (.02–3.40)	n/a
	*06:04:01*	.22 (.05–1.34)	n/a	n/a	.40 (.06–2.95)
	*06:09*	**.16 (.05**–**.56)**	.30 (.05–1.88)	n/a	.32 (.05–2.04)
HLA-DRB1*	*01:02:01*			.16 (.02–1.61)	
	*03:01:01*			n/a	
	*03:02:01*			.35 (.03–3.66)	
	*07:01:01*			.10 (.01–1.06)	
	*09:01:02*			.25 (.02–2.62)	
	*11:01*			.17 (.02–1.28)	
	*11:02:01*			n/a	
	*12:01*			n/a	
	*13:01:01*			.14 (.01–1.39)	
	*13:02:01*			n/a	
	*15:03*			**.08 (.01**–**.59)**	

Only loci/phenotype associations determined as significant in Table 2 are included. Results in bold are those allele/phenotypes associations where the 95% confidence interval for the odds ratio excludes 1.

Abbreviations: CI, confidence interval; DILI, drug-induced liver injury; HLA, human leukocyte antigen; HSR, hypersensitivity reaction; HSS, hypersensitivity syndrome; n/a, odds ratios were not possible to calculate due to the low frequency of the allele in that phenotype; SJS, Stevens-Johnson syndrome; TEN, toxic epidermal necrolysis.

Our analysis showed that HLA-C*04:01 predisposed to nevirapine hypersensitivity. Individuals who carry HLA-C*04:01 were at higher risk of developing hypersensitivity reactions in general (OR = 2.64 [95% CI, 1.13–2.64]), and, specifically SJS/TEN (OR = 17.52 [95% CI, 3.31–92.80]) when exposed to nevirapine than were carriers of the “rare alleles,” the most common group of HLA alleles in C locus. This association was not observed with any other phenotype.

Multilocus haplotypes for class I and II *HLA* loci were generated to determine the structure of haplotypes across multiple loci in our cohort from Malawi (Table 4). The data suggest high linkage disequilibrium between the *HLA-B* and *C* loci in both the nevirapine hypersensitive and tolerant patients (D′ = 0.946 and 0.924, respectively). Significant linkage disequilibrium was observed in the hypersensitive and tolerant groups between the DQB1 and DRB1 loci (D′ = 0.890 and 0.898 respectively). Haplotype frequencies were calculated for 5-loci haplotypes and for combinations of *HLA-B*, *C*, *DRB1*, and *DQB1* loci haplotypes containing the HLA-C*04:01 allele (Table 5 and Supplementary Table 2). The frequency of the HLA B53:01:01/C*04:01 haplotype was significantly higher in the hypersensitive cohort (0.121) than the tolerant group (0.039). There was no difference in *DRB1/DQB1* haplotype frequencies in haplotypes containing the DQB1*05:01:01 allele (Table 5).

**Table 4.** Linkage Disequilibrium Analysis of Class I and II Human Leukocyte Antigen Loci in Nevirapine-Hypersensitive and -Tolerant Malawian Cohorts

	Locus Pair	D′	
		All HSR	Tolerant
1	A|B	0.747	0.746
2	A|C	0.659	0.666
3	A|DRB1	0.642	0.621
4	A|DQB1	0.599	0.523
5	B|C	0.946	0.924
6	B|DRB1	0.760	0.743
7	B|DQB1	0.650	0.592
8	C|DRB1	0.632	0.635
9	C|DQB1	0.581	0.538
10	DRB1|DQB1	0.890	0.898

Data represent the D′ value for each pairwise analysis as determined by PyPop 0.7.0 software.

Abbreviation: HSR, hypersensitivity reaction.

**Table 5.** Frequency of Human Leukocyte Antigen Haplotype Frequencies in the Nevirapine-Hypersensitive and -Tolerant Cohorts

	Hypersensitive (n = 116)		Tolerant (n = 153)	
	Frequency	Counts	Frequency	Counts
*B*|*C* Haplotype				
* 53:01:01|04:01*	0.121	26	0.039	12
* 44:03|04:01*	0.069	16	0.059	18
* 35:01|04:01*	0.026	6	0.023	7
* 15:10|04:01*	0.013	3	0.006	2
* 58:02|04:01*	0.004	1		
* 15:03|04:01*	0.004	1		
* 37:01:01|04:01*	0.004	1		
* 81:01|04:01*	0.004	1		
* 42:01|04:01*			0.003	1
* 57:01:01|04:01*			0.006	2
* 58:01|04:01*			0.003	1
	Cases (n = 93)		Controls (n = 89)	
*DRB1|DQB1* Haplotype				
* 01:02:01|05:01:01*	0.038	7	0.073	13
* 12:01|05:01:01*	0.038	7	0.062	11
* 13:01:01|05:01:01*	0.032	6	0.028	5
* 01:01:01|05:01:01*	0.011	2	0.006	1
* 10:01|05:01:01*	0.011	2	0.011	2
* 13:02:01|05:01:01*	0.005	1		
* 14:01|05:01:01*			0.006	1
* 15:03|05:01:01*			0.006	1

Only B|C haplotypes containing the C*04:01 and *DRB1\DQB1* haplotypes containing DQB1*05:01:01 are listed. Frequency data for class I and class II haplotypes are listed in Supplementary Table 2.

Subsequent analysis was undertaken incorporating all *HLA*-typed individuals to determine the absolute risk of SJS/TEN and predictive values of HLA-C*04:01, the HLA-B*53:01:01/C*04:01 haplotype, and DQB1*05:0101 carriage (Table 6). The OR for overall risk of SJS/TEN associated with HLA-C*04:01 was 5.17 (95% CI, 2.39–11.18; *P* < .0001). Positive predictive values (PPVs) and negative predictive values (NPVs), based on a SJS/TEN prevalence of 1.07%, were 2.6% and 99.2%, respectively. The OR for overall risk of SJS/TEN associated with carriage of the *HLA*-B*53:01:01/C*04:01 haplotype (5.17 [95% CI, 1.83–14.28]) was comparable to HLA-C*04:01 alone, although the NPV was lower (91.6%). For DQB*05:0101 the OR was 0.17 (95% CI, .05–.60), and the PPV and NPV were 0.3% and 98.6%, respectively. Other *HLA* allele/hypersensitivity phenotype associations noted in Table 3 demonstrated PPVs between 0.08% and 3.1% and NPVs between 22.3% and 36.2% (data not shown).

**Table 6.** Predictive Value for Stevens-Johnson Syndrome/Toxic Epidermal Necrolysis Risk of HLA-C*04:01, B*53:01:01|*04:01, and DQB1*05:01:01 Carriage

	SJS/TEN	Tolerant	Total	
HLA-C*04:01				
Positive	23	39	62	PPV = 2.6%	
Negative	13	114	127	NPV = 99.2%
All	36	153		
	Sensitivity = 63.9%	Specificity = 74.4%	OR = 5.17 (95% CI, 2.39–11.18), *P* < .0001	
HLA-B*53:01:01|HLA-C*04:01				
Positive	11	12	33	PPV = 4.2%
Negative	25	141	176	NPV = 91.6%
All	36	153		
	Sensitivity = 31.4%	Specificity = 92.2%	OR = 5.17 (95% CI, 1.83–14.28), *P* = .0002	
HLA-DQB1*05:01:01				
Positive	3	47	50	PPV = 0.3%
Negative	32	88	120	NPV = 98.6%
All	35	135		
	Sensitivity = 0.9%	Specificity = 65.1%	OR = 0.17 (95% CI, .05–.60), *P* = .0024	

Positive and negative predictive values as well as sensitivity and specificity are shown. Prevalence of SJS/TEN is assumed at 1.07% based on an incidence of 12 of 1117 observed in the prospective study. Odds ratio with 95% confidence and *P* value are determined using a 2 × 2 χ^2^ test.

Abbreviations: CI, confidence interval; HLA, human leukocyte antigen; NPV, negative predictive value; OR, odds ratio; PPV, positive predictive value; SJS, Stevens-Johnson syndrome; TEN, toxic epidermal necrolysis.

## DISCUSSION

Nevirapine-induced hypersensitivity reactions have shown an association with a number of *HLA* alleles (Table 1), which vary according to ethnicity and the phenotype of the reaction [23]. The main finding of the present study is that *HLA*-C*04:01 predisposes to nevirapine-cutaneous reactions with the greatest risk observed with SJS/TEN (OR = 5.17 [95% CI, 2.39–11.18]; Table 3), the severest form of hypersensitivity in terms of mortality [24–26]. The risk associated with *HLA*-B*53:01:01/C*04:01 haplotype carriage was comparable. Its sensitivity as a biomarker for SJS/TEN was 31.4%, compared to 63.9% for HLA-C*04:01 alone (Table 6), suggesting that the association is driven by carriage of 1 allele at a single *HLA* locus. This is supported by the haplotype analysis of the *HLA* loci in this particular Malawian population (Table 4). Although HLA-C*04 (along with B*35) has been associated with the development of AIDS in whites [27], no association between HLA-C*04 and HIV has been reported in African populations or any other ethnic group. This is the first report of an association between nevirapine-induced SJS/TEN and *HLA*-C*04:01, but is consistent with previous studies in black African (OR = 5.17) [14], Thai (OR = 3.79) [15], and Chinese (OR = 3.23) [16] populations that have reported an association with HLA-C*04 nevirapine-cutaneous reactions. A meta-analysis of our data, related to HLA-C*04:01 carriage and cutaneous nevirapine hypersensitivity reactions (n = 102), but excluding patients who had DILI only (n = 15), with the only eligible previous study in a black American population (OR = 5.17 [95% CI, 1.81–14.78]) [14] gave a combined OR of 3.34 (95% CI, 1.60–4.98) (Supplementary Figure 1).

Although it would be useful to replicate in other populations, the data available to date strongly suggest that *HLA*-C*04:01 predisposes to nevirapine-induced cutaneous reactions of different severities (including SJS/TEN) in several ethnic groups. The predictive value of HLA-C*04:01 as a biomarker of nevirapine-induced SJS/TEN is limited (Table 4). The incidence of nevirapine-induced SJS/TEN in our prospective study was 12 of 1117 patients (1.07%). This gives a PPV of 2.6%, which is of no diagnostic value. Although the NPV is 99.2%, it does not reach 100%, which has been recommended [23]. It is important to note that in our population, HLA-C*04:01 was associated with SJS/TEN, which is almost always drug-related, severe, and perhaps more easily recognizable than other drug-induced adverse phenotypes. This contrasts with abacavir hypersensitivity, which varies in severity and can be more difficult to differentiate from other causes. This is reflected in the fact that the NPV of HLA-B*57:01 was 95.5% for clinically diagnosed abacavir-induced hypersensitivity and 100% for immunologically confirmed abacavir hypersensitivity [28].

We did not replicate a previous associations between HLA-DRB1*01:01 and nevirapine-induced hypersensitivity [12–14] in whites. This is possibly due to ethnic differences in the frequency of *HLA* alleles. HLA-DRB1*01:01 was observed at a low frequency in our Malawian cohort (0.008) with 1 tolerant and 2 hypersensitive carriers out of 182 individuals genotyped.

The observation of an apparent protective effect of 6 different *HLA-DQB1* (Table 3) across a number of different phenotypes is interesting. A number studies have identified protective *HLA* alleles (Table 1), but there is no common pattern. These may not represent the actual protective alleles given the high degree of linkage disequilibrium across the MHC. Further work in larger populations is needed to elucidate the interaction between risk and protective *HLA* alleles in predisposing to different forms of nevirapine hypersensitivity. Of note here is that the pathogenesis of nevirapine hypersensitivity is immune-mediated, as shown by a positive lymphocyte transformation test in a patient with DILI [29]. However the mechanism by which this occurs is unknown. Three possible hypotheses have been suggested, including the hapten hypothesis [30], pharmacological interaction hypothesis [31], and altered peptide binding profile [32]. It is possible that based on the *HLA* profile of an individual, the interaction between nevirapine (and its antigen) and the HLA molecules leads to either a protective or a predisposing effect. Such an allele-competing effect has been postulated for the HLA-associated disease narcolepsy [33].

Studies have also evaluated the role of CYP2B6, which metabolizes nevirapine, in predisposing to hypersensitivity. CYPB6 shows wide interindividual variability in expression and activity in human livers [34]. It contains a functional exonic variant (c.516G>T), which causes loss of enzymatic function [35]; is associated with higher plasma concentrations in black and white populations [36, 37]; and has been associated with nevirapine-induced cutaneous adverse reactions [14] and neuropsychological toxicity [36], though not hepatotoxicity [38]. The combination of c.516G>T and HLA-Cw*04 alleles showed a stronger association in black, white, and Asian populations than c.516 G>T alone [14]. In our cohort, however, the *CYP2B6* c.516G>T polymorphism was not a significant risk factor for any of the hypersensitivity phenotypes (data not shown); therefore, a combination analysis has not been undertaken.

Our study has several strengths: (1) we investigated a Malawian HIV cohort originating from a highly homogeneous population from a small geographic area; thus, the effect of ethnicity admixture is likely to be minimal; (2) sex- and age-matching of our tolerant controls also minimized the effect of these nongenetic factors; (3) when compared to previous studies, our sample size was larger; (4) we used strict phenotypic characterization with independent adjudication by a dermatologist; (5) the majority of patients were recruited prospectively where detailed phenotypic data could be gathered, although we did include 28 retrospectively identified patients; (6) close monitoring of patients with nonsevere rash, treated through and excluded as cases, further strengthened the phenotype by omitting potential false positives; and (7) we used sequence-based *HLA* typing to at least 4 digits, which is particularly important in this population because of the presence of some rare alleles. However, there are also some limitations. First, despite a large sample size, when subdividing the groups into phenotypes, the numbers within categories fell, limiting our power to detect true associations. This is a recognized drawback of studying nevirapine hypersensitivity, where the phenotypic manifestations not only vary, but have different allelic associations (Table 1). Second, we could not genotype all patients, particularly for the class II *HLA* alleles, which would have strengthened haplotype analysis, nevertheless, our study was larger than previous studies despite the missing data. Third, given the homogeneity of the Malawian population, it is possible that although the *HLA* association identified here is relevant, it may not be applicable to other ethnicities including other African populations.

In conclusion, we have identified an association between the HLA-C*04:01 allele nevirapine-induced hypersensitivity phenotypes, including the first report of an association between HLA-C*04:01 and the most severe phenotype, SJS/TEN. Our study appears to replicate previous observations [14] of an association between HLA-C*04:01 and risk of nevirapine cutaneous adverse drug reactions in a black population. Further work is required to replicate the association identified here, and to evaluate in more detail the effects of risk and competing HLA alleles. Additionally, functional in vitro or in silico models are needed to clarify the mechanisms of the immune-mediated response to nevirapine and its metabolites [39].

## Supplementary Data

Supplementary materials are available at *Clinical Infectious Diseases* online (http://cid.oxfordjournals.org/). Supplementary materials consist of data provided by the author that are published to benefit the reader. The posted materials are not copyedited. The contents of all supplementary data are the sole responsibility of the authors. Questions or messages regarding errors should be addressed to the author.

Supplementary Data

## References

[1] van Leth F, Phanuphak P, Ruxrungtham K (2004). Comparison of first-line antiretroviral therapy with regimens including nevirapine, efavirenz, or both drugs, plus stavudine and lamivudine: a randomised open-label trial, the 2NN Study. Lancet.

[2] Siegfried NL, Van Deventer PJ, Mahomed FA, Rutherford GW (2006). Stavudine, lamivudine and nevirapine combination therapy for treatment of HIV infection and AIDS in adults. Cochrane Database Syst Rev.

[3] Phillips E, Gutierrez S, Jahnke N (2007). Determinants of nevirapine hypersensitivity and its effect on the association between hepatitis C status and mortality in antiretroviral drug-naive HIV-positive patients. AIDS.

[4] Wit FW, Kesselring AM, Gras L (2008). Discontinuation of nevirapine because of hypersensitivity reactions in patients with prior treatment experience, compared with treatment-naive patients: the ATHENA cohort study. Clin Infect Dis.

[5] Fagot JP, Mockenhaupt M, Bouwes-Bavinck JN, Naldi L, Viboud C, Roujeau JC (2001). Nevirapine and the risk of Stevens-Johnson syndrome or toxic epidermal necrolysis. AIDS.

[6] Mittmann N, Knowles SR, Koo M, Shear NH, Rachlis A, Rourke SB (2012). Incidence of toxic epidermal necrolysis and Stevens-Johnson syndrome in an HIV cohort: an observational, retrospective case series study. Am J Clin Dermatol.

[7] De Maat MM, Mathot RA, Veldkamp AI (2002). Hepatotoxicity following nevirapine-containing regimens in HIV-1-infected individuals. Pharmacol Res.

[8] Mallal S, Nolan D, Witt C (2002). Association between presence of HLA-B*5701, HLA-DR7, and HLA-DQ3 and hypersensitivity to HIV-1 reverse-transcriptase inhibitor abacavir. Lancet.

[9] Chung WH, Hung SI, Hong HS (2004). Medical genetics: a marker for Stevens-Johnson syndrome. Nature.

[10] McCormack M, Alfirevic A, Bourgeois S (2011). HLA-A*3101 and carbamazepine-induced hypersensitivity reactions in Europeans. N Engl J Med.

[11] Hung SI, Chung WH, Liou LB (2005). HLA-B*5801 allele as a genetic marker for severe cutaneous adverse reactions caused by allopurinol. Proc Natl Acad Sci U S A.

[12] Martin AM, Nolan D, James I (2005). Predisposition to nevirapine hypersensitivity associated with HLA-DRB1*0101 and abrogated by low CD4 T-cell counts. AIDS.

[13] Vitezica ZG, Milpied B, Lonjou C (2008). HLA-DRB1*01 associated with cutaneous hypersensitivity induced by nevirapine and efavirenz. AIDS.

[14] Yuan J, Guo S, Hall D (2011). Toxicogenomics of nevirapine-associated cutaneous and hepatic adverse events among populations of African, Asian, and European descent. AIDS.

[15] Likanonsakul S, Rattanatham T, Feangvad S (2009). HLA-Cw*04 allele associated with nevirapine-induced rash in HIV-infected Thai patients. AIDS Res Ther.

[16] Gao S, Gui XE, Liang K, Liu Z, Hu J, Dong B (2011). HLA-dependent hypersensitivity reaction to nevirapine in Chinese Han HIV-infected patients. AIDS Res Hum Retroviruses.

[17] Gatanaga H, Yazaki H, Tanuma J (2007). HLA-Cw8 primarily associated with hypersensitivity to nevirapine. AIDS.

[18] Chantarangsu S, Mushiroda T, Mahasirimongkol S (2009). HLA-B*3505 allele is a strong predictor for nevirapine-induced skin adverse drug reactions in HIV-infected Thai patients. Pharmacogenet Genomics.

[19] Garcia-Cortes M, Lucena MI, Pachkoria K, Borraz Y, Hidalgo R, Andrade RJ (2008). Evaluation of Naranjo adverse drug reactions probability scale in causality assessment of drug-induced liver injury. Aliment Pharmacol Ther.

[20] Naranjo CA, Fornazzari L, Sellers EM (1981). Clinical detection and assessment of drug induced neurotoxicity. Prog Neuropsychopharmacol.

[21] French LE (2006). Toxic epidermal necrolysis and Stevens Johnson syndrome: our current understanding. Allergol Int.

[22] Lancaster A, Nelson MP, Meyer D, Thomson G, Single RM (2003). PyPop: a software framework for population genomics: analyzing large-scale multi-locus genotype data. Pac Symp Biocomput.

[23] Pavlos R, Mallal S, Phillips E (2012). HLA and pharmacogenetics of drug hypersensitivity. Pharmacogenomics.

[24] Chan HL, Stern RS, Arndt KA (1990). The incidence of erythema multiforme, Stevens-Johnson syndrome, and toxic epidermal necrolysis. A population-based study with particular reference to reactions caused by drugs among outpatients. Arch Dermatol.

[25] Roujeau JC, Chosidow O, Saiag P, Guillaume JC (1990). Toxic epidermal necrolysis (Lyell syndrome). J Am Acad Dermatol.

[26] Schopf E, Stuhmer A, Rzany B, Victor N, Zentgraf R, Kapp JF (1991). Toxic epidermal necrolysis and Stevens-Johnson syndrome. An epidemiologic study from West Germany.. Arch Dermatol.

[27] Carrington M, Nelson GW, Martin MP (1999). HLA and HIV-1: heterozygote advantage and B*35-Cw*04 disadvantage. Science.

[28] Mallal S, Phillips E, Carosi G (2008). HLA-B*5701 screening for hypersensitivity to abacavir. N Engl J Med.

[29] Drummond NS, Vilar FJ, Naisbitt DJ (2006). Drug-specific T cells in an HIV-positive patient with nevirapine-induced hepatitis. Antiviral Ther.

[30] Uetrecht J (2007). Idiosyncratic drug reactions: current understanding. Annu Rev Pharmacol Toxicol.

[31] Pichler WJ, Beeler A, Keller M (2006). Pharmacological interaction of drugs with immune receptors: the p-i concept. Allergol Int.

[32] Ostrov DA, Grant BJ, Pompeu YA (2012). Drug hypersensitivity caused by alteration of the MHC-presented self-peptide repertoire. Proc Natl Acad Sci U S A.

[33] Han F, Lin L, Li J (2012). HLA-DQ association and allele competition in Chinese narcolepsy. Tissue Antigens.

[34] Code EL, Crespi CL, Penman BW, Gonzalez FJ, Chang TK, Waxman DJ (1997). Human cytochrome P4502B6: interindividual hepatic expression, substrate specificity, and role in procarcinogen activation. Drug Metab Dispos.

[35] Lang T, Klein K, Fischer J (2001). Extensive genetic polymorphism in the human CYP2B6 gene with impact on expression and function in human liver. Pharmacogenetics.

[36] Rotger M, Colombo S, Furrer H (2005). Influence of CYP2B6 polymorphism on plasma and intracellular concentrations and toxicity of efavirenz and nevirapine in HIV-infected patients. Pharmacogenet Genomics.

[37] Penzak SR, Kabuye G, Mugyenyi P (2007). Cytochrome P450 2B6 (CYP2B6) G516T influences nevirapine plasma concentrations in HIV-infected patients in Uganda. HIV Med.

[38] Haas DW, Bartlett JA, Andersen JW (2006). Pharmacogenetics of nevirapine-associated hepatotoxicity: an adult AIDS Clinical Trials Group collaboration. Clin Infect Dis.

[39] Srivastava A, Lian LY, Maggs JL (2010). Quantifying the metabolic activation of nevirapine in patients by integrated applications of NMR and mass spectrometries. Drug Metab Dispos.

[40] Littera R, Carcassi C, Masala A (2006). HLA-dependent hypersensitivity to nevirapine in Sardinian HIV patients. AIDS.

[41] Umapathy S, Pawar A, Bajpai S, Pazare AR, Ghosh K (2011). HLA involvement in nevirapine-induced dermatological reaction in antiretroviral-treated HIV-1 patients. J Pharmacol Pharmacother.

