# Supplementary material for: Association of Human Leukocyte Antigen Alleles and Nevirapine Hypersensitivity in a Malawian HIV-Infected Population
Source: Clin Infect Dis. 2013 Jan 29;56(9):1330–9. doi: 10.1093/cid/cit021 (PMC3616517; doi:10.1093/cid/cit021)
Supplement: Supplementary Data [file supp_56_9_1330__index.html]

Association of Human Leukocyte Antigen Alleles and Nevirapine Hypersensitivity in a Malawian HIV-Infected Population — Association of Human Leukocyte Antigen Alleles and Nevirapine Hypersensitivity in a Malawian HIV-Infected Population — Association of Human Leukocyte Antigen Alleles and Nevirapine Hypersensitivity in a Malawian HIV-Infected Population — Supplementary Data 

# Association of Human Leukocyte Antigen Alleles and Nevirapine Hypersensitivity in a Malawian HIV-Infected Population

## Supplementary Data

Supplementary Data

**Files in this Data Supplement:**

- Supplementary Data - Doc file
