# Supplementary material for: Association of Human Leukocyte Antigen Alleles and Nevirapine Hypersensitivity in a Malawian HIV-Infected Population
Source: Clin Infect Dis. 2013 Jan 29;56(9):1330–9. doi: 10.1093/cid/cit021 (PMC3616517; doi:10.1093/cid/cit021)

**SUPPLEMENTARY TABLES**

**Supplementary Table 1.** *HLA-A, B* and *C*, *DQQB1 and DRB1* allele frequencies in a Malawian HIV population receiving nevirapine. Data is indicative of allele frequencies observed in nevirapine-tolerant and intolerant patient cohorts. “Rare” allele represents an amalgamation of all alleles of that specific loci where frequency was <0.02. G denotes allele types with ambiguity call at higher resolution which were merged together.

| ***HLA-A**** | **Carriage Frequency** | | | | | |
| --- | --- | --- | --- | --- | --- | --- |
|  | **All Cases (n=117)** | **Controls (n=153)^6^** | **DILI^1^ (n=18)** | **SJS^1^ (=36)** | **HSS (n=20)** | **NIR (n=46)** |
| ***01:01*** | 0.04 | 0.04 | 0.00 | 0.00 | 0.05 | 0.09 |
| ***02:01*** | 0.16 | 0.16 | 0.11 | 0.22 | 0.30 | 0.09 |
| ***02:05*** | 0.08 | 0.05 | 0.17 | 0.06 | 0.10 | 0.07 |
| ***03:01*** | 0.08 | 0.07 | 0.06 | 0.17 | 0.00 | 0.07 |
| ***23:01*** | 0.19 | 0.20 | 0.06 | 0.11 | 0.30 | 0.24 |
| ***29:02:01*** | 0.15 | 0.10 | 0.28 | 0.14 | 0.15 | 0.09 |
| ***30:01*** | 0.16 | 0.20 | 0.11 | 0.17 | 0.25 | 0.13 |
| ***30:02*** | 0.24 | 0.24 | 0.17 | 0.19 | 0.25 | 0.28 |
| ***34:02*** | 0.12 | 0.06 | 0.11 | 0.22 | 0.10 | 0.04 |
| ***36:01*** | 0.10 | 0.10 | 0.06 | 0.11 | 0.15 | 0.09 |
| ***66:01*** | 0.08 | 0.09 | 0.06 | 0.00 | 0.00 | 0.17 |
| ***68:01*** | 0.06 | 0.06 | 0.06 | 0.00 | 0.10 | 0.09 |
| ***68:02*** | 0.18 | 0.20 | 0.28 | 0.28 | 0.00 | 0.15 |
| ***74:01*** | 0.06 | 0.14 | 0.11 | 0.06 | 0.05 | 0.04 |
| ***rare*** | 0.21 | 0.22 | 0.33 | 0.22 | 0.10 | 0.24 |
|  |  |  |  |  |  |  |
| ***HLA-B**** | **Carriage Frequency** | | | | | |
|  | **All Cases (n=117)** | **Controls (n=153)^6^** | **DILI^1^ (n=18)** | **SJS^1^ (=36)** | **HSS (n=20)** | **NIR (n=46)** |
| ***07:02*** | 0.06 | 0.08 | 0.11 | 0.06 | 0.05 | 0.04 |
| ***08:01*** | 0.06 | 0.04 | 0.00 | 0.08 | 0.00 | 0.09 |
| ***14:01*** | 0.04 | 0.12 | 0.00 | 0.06 | 0.00 | 0.07 |
| ***14:02:01*** | 0.06 | 0.06 | 0.06 | 0.06 | 0.00 | 0.09 |
| ***15:03*** | 0.12 | 0.20 | 0.28 | 0.11 | 0.10 | 0.09 |
| ***15:10*** | 0.12 | 0.10 | 0.06 | 0.17 | 0.10 | 0.13 |
| ***18:01*** | 0.07 | 0.07 | 0.06 | 0.08 | 0.05 | 0.09 |
| ***35:01*** | 0.05 | 0.04 | 0.06 | 0.03 | 0.00 | 0.09 |
| ***39:10*** | 0.04 | 0.04 | 0.00 | 0.06 | 0.00 | 0.07 |
| ***42:01*** | 0.15 | 0.16 | 0.17 | 0.17 | 0.20 | 0.13 |
| ***44:03*** | 0.17 | 0.16 | 0.06 | 0.28 | 0.15 | 0.13 |
| ***45:01*** | 0.09 | 0.12 | 0.17 | 0.06 | 0.15 | 0.04 |
| ***53:01:01*** | 0.25 | 0.10 | 0.17 | 0.31 | 0.20 | 0.24 |
| ***57:03:01*** | 0.04 | 0.05 | 0.06 | 0.00 | 0.10 | 0.04 |
| ***58:01*** | 0.14 | 0.11 | 0.33 | 0.11 | 0.15 | 0.09 |
| ***58:02*** | 0.09 | 0.14 | 0.00 | 0.06 | 0.00 | 0.17 |
| ***81:01*** | 0.09 | 0.08 | 0.00 | 0.08 | 0.10 | 0.11 |
| ***rare*** | 0.25 | 0.24 | 0.39 | 0.19 | 0.40 | 0.17 |
|  |  |  |  |  |  |  |
| ***HLA-C*** | **Carriage Frequency** | | | | | |
|  | **All Cases (n=117)** | **Controls (n=153)^6^** | **DILI^1^ (n=18)** | **SJS (=36)** | **HSS (n=20)** | **NIR (n=46)** |
| ***02:10*** | 0.11 | 0.24 | 0.28 | 0.11 | 0.10 | 0.07 |
| ***03:02*** | 0.03 | 0.05 | 0.00 | 0.03 | 0.05 | 0.04 |
| ***03:03*** | 0.04 | 0.04 | 0.00 | 0.03 | 0.05 | 0.07 |
| ***03:04:02*** | 0.09 | 0.08 | 0.06 | 0.14 | 0.10 | 0.09 |
| ***04:01*** | 0.46 | 0.25 | 0.22 | 0.64 | 0.40 | 0.41 |
| ***06:02*** | 0.14 | 0.24 | 0.22 | 0.08 | 0.10 | 0.15 |
| ***07:01*** | 0.26 | 0.16 | 0.50 | 0.25 | 0.30 | 0.17 |
| ***07:02*** | 0.06 | 0.08 | 0.11 | 0.06 | 0.05 | 0.04 |
| ***07:04*** | 0.05 | 0.05 | 0.00 | 0.06 | 0.05 | 0.07 |
| ***08:02*** | 0.11 | 0.13 | 0.11 | 0.11 | 0.05 | 0.13 |
| ***12:03*** | 0.05 | 0.04 | 0.00 | 0.06 | 0.00 | 0.09 |
| ***16:01:01*** | 0.08 | 0.10 | 0.06 | 0.08 | 0.10 | 0.07 |
| ***17:01*** | 0.21 | 0.21 | 0.22 | 0.19 | 0.30 | 0.17 |
| ***18:01*** | 0.11 | 0.14 | 0.06 | 0.08 | 0.15 | 0.13 |
| ***rare*** | 0.09 | 0.13 | 0.17 | 0.06 | 0.05 | 0.11 |
|  |  | | | | | |
| ***HLA-DQB1**** | **Carriage Frequency** | | | | | |
|  | **All Cases (n=106)^2^** | **Controls (n=135)^3^** | **DILI^1^ (n=14)** | **SJS^1^ (=35)** | **HSS (n=20)** | **NIR (n=40)** |
| ***02:01G*** | 0.34 | 0.36 | 0.14 | 0.43 | 0.30 | 0.38 |
| ***03:01G*** | 0.31 | 0.15 | 0.50 | 0.31 | 0.30 | 0.28 |
| ***03:02:01*** | 0.04 | 0.06 | 0.07 | 0.06 | 0.00 | 0.03 |
| ***04:02*** | 0.12 | 0.11 | 0.07 | 0.11 | 0.25 | 0.10 |
| ***05:01:01*** | 0.25 | 0.35 | 0.43 | 0.09 | 0.25 | 0.35 |
| ***06:02*** | 0.50 | 0.57 | 0.29 | 0.66 | 0.35 | 0.48 |
| ***06:03:01*** | 0.05 | 0.10 | 0.07 | 0.06 | 0.00 | 0.05 |
| ***06:04:01*** | 0.04 | 0.05 | 0.00 | 0.00 | 0.10 | 0.05 |
| ***06:09*** | 0.05 | 0.11 | 0.00 | 0.06 | 0.10 | 0.03 |
| ***rare*** | 0.08 | 0.07 | 0.07 | 0.06 | 0.10 | 0.10 |
|  |  |  |  |  |  |  |
| ***HLA-DRB1**** | **Carriage Frequency** | | | | | |
|  | **All Cases (n=93)^4^** | **Controls (n=89)^5^** | **DILI^1^ (n=14)** | **SJS^1^ (=31)** | **HSS (n=17)** | **NIR (n=34)** |
| ***01:02:01*** | 0.08 | 0.13 | 0.14 | 0.06 | 0.12 | 0.06 |
| ***03:01:01*** | 0.13 | 0.11 | 0.00 | 0.19 | 0.06 | 0.15 |
| ***03:02:01*** | 0.10 | 0.08 | 0.07 | 0.10 | 0.12 | 0.12 |
| ***07:01:01*** | 0.14 | 0.18 | 0.07 | 0.16 | 0.12 | 0.18 |
| ***09:01:02*** | 0.05 | 0.10 | 0.07 | 0.06 | 0.12 | 0.03 |
| ***11:01*** | 0.33 | 0.27 | 0.29 | 0.35 | 0.35 | 0.32 |
| ***11:02:01*** | 0.13 | 0.07 | 0.29 | 0.10 | 0.12 | 0.12 |
| ***12:01*** | 0.08 | 0.12 | 0.00 | 0.03 | 0.00 | 0.18 |
| ***13:01:01*** | 0.18 | 0.21 | 0.21 | 0.13 | 0.18 | 0.21 |
| ***13:02:01*** | 0.08 | 0.10 | 0.00 | 0.06 | 0.18 | 0.06 |
| ***15:03*** | 0.34 | 0.44 | 0.21 | 0.42 | 0.29 | 0.32 |
| ***rare*** | 0.22 | 0.13 | 0.43 | 0.16 | 0.18 | 0.18 |

**Footnote:** ^1^=3 patients had both DILI and SJS; ^2^= genotype missing for 11 patients, ^3^= genotype missing for 19 patients, ^4^= genotype missing for 24 patients, ^5^= genotype missing for 65 patients, ^6^= genotype missing for 1 patient

**Supplementary Table 2**. Counts and frequencies of the three most common A:B:C:DRB1:DQB1, B:C:DRB1 and C:DRB1:DQB1 haplotypes containing the C*04:01 allele in nevirapine-hypersensitive cases and nevirapine-tolerant controls.

| **HLA-A** | **HLA-B** | **HLA-C** | **HLA-DRB1** | **HLA-DQB1** | **Cases (%)** | **Controls (%)** |
| --- | --- | --- | --- | --- | --- | --- |
|  | | | | | | |
| 36:01 | 53:01:01 | 04:01 | 11:01 | 06:02 | 6 (0.065) | 2 (0.023) |
| 34:02 | 44:03 | 04:01 | 15:03 | 06:02 | 4 (0.043) | 2 (0.023) |
| 23:01 | 44:03 | 04:01 | 15:03 | 06:02 | 3 (0.033) | 4 (0.045) |
|  | | | | | | |
| any | 44:03 | 04:01 | 15:03 | any | 8 (0.087) | 7 (0.08) |
| any | 53:01:01 | 04:01 | 11:01 | any | 8 (0.087) | 3 (0.034) |
| any | 53:01:01 | 04:01 | 08:04 | any | 4 (0.043) | 0 |
|  | | | | | | |
| any | any | 04:01 | 15:03 | 06:02 | 16 (0.17) | 7 (0.08) |
| any | any | 04:01 | 11:01 | 06:02 | **9 (0.098)** | **1 (0.011)** |
| any | any | 04:01 | 07:01 | 02:01 | 4 (0.043) | 1 (0.011) |

- Haplotype frequencies were derived from complete HLA genotype data in hypersensitive cases (N=92) and tolerant controls (N=88).
- One haplotype (C*04:01 DRB1*11:01 DQB1*06:02) is present at a higher frequency in the hypersensitive group than in tolerant controls (OR 9.4, 95%CI 1.2 to 418.1, p=0.018). However, after correction for multiple comparisons given the number of different C:DRB1:DQB1 haplotypes estimated in cases (N=342) and in controls (N=371), the difference is not statistically significant. The total number of possible five loci haplotypes estimated using the EM algorithm in PyPop is N=2139 in cases and N=2262 in tolerant controls.

**SUPLEMENTARY FIGURE LEGENDS**

**Supplementary Figure 1.** Odds ratio meta-analysis of all cutaneous nevirapine-induced ADRs in our Malawian HIV cohort with data from previously published association in a black HIV cohort . Size of squares denotes weighting of study in the analysis. Diamond indicates the pooled odds ratio and horizontal lines indicate the 95% confidence interval**.**


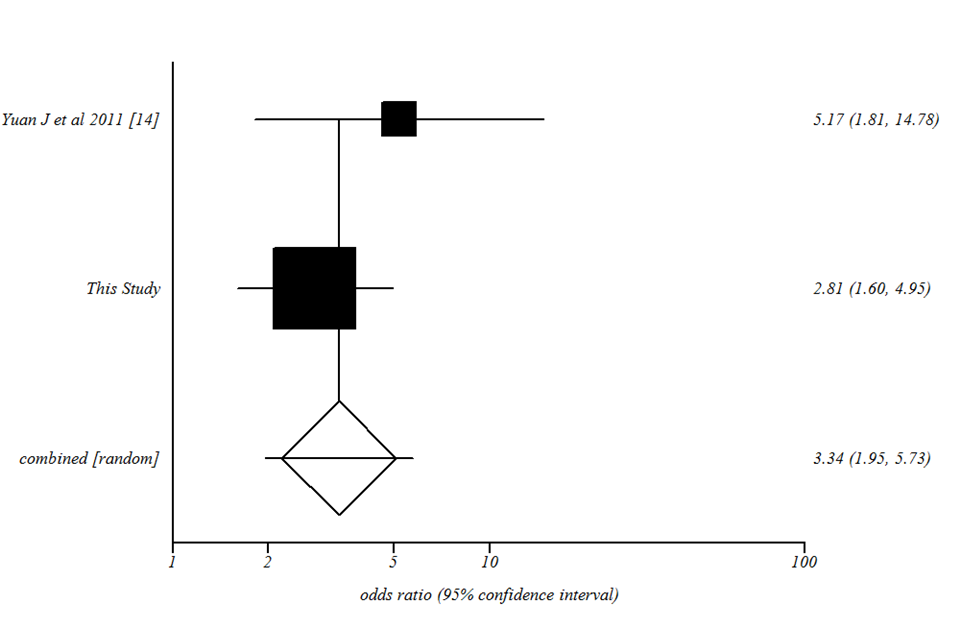

Supplement: Supplementary Data [file supp_cit021_cit021supp.doc]
